# MLG-STPM: Meta-Learning Guided STPM for Robust Industrial Anomaly Detection Under Label Noise

**DOI:** 10.3390/s25196255

**Published:** 2025-10-09

**Authors:** Yu-Hang Huang, Sio-Long Lo, Zhen-Qiang Chen, Jing-Kai Wang

**Affiliations:** Faculty of Innovation Engineering, Macau University of Science and Technology, Macau 999078, China; 3240000285@student.must.edu.mo (Y.-H.H.); 3220002509@student.must.edu.mo (Z.-Q.C.); ming359046415@gmail.com (J.-K.W.)

**Keywords:** anomaly detection, industrial image analysis, label noise, student–teacher learning, meta-learning, deep learning

## Abstract

Industrial image anomaly detection (IAD) is crucial for quality control, but its performance often degrades when training data contain label noise. To circumvent the reliance on potentially flawed labels, unsupervised methods that learn from the data distribution itself have become a mainstream approach. Among various unsupervised techniques, student–teacher frameworks have emerged as a highly effective paradigm. Student–Teacher Feature Pyramid Matching (STPM) is a powerful method within this paradigm, yet it is susceptible to such noise. Inspired by STPM and aiming to solve this issue, this paper introduces Meta-Learning Guided STPM (MLG-STPM), a novel framework that enhances STPM’s robustness by incorporating a guidance mechanism inspired by meta-learning. This guidance is achieved through an Evolving Meta-Set (EMS), which dynamically maintains a small high-confidence subset of training samples identified by their low disagreement between student and teacher networks. By training the student network on a combination of the current batch and the EMS, MLG-STPM effectively mitigates the impact of noisy labels without requiring an external clean dataset or complex re-weighting schemes. Comprehensive experiments on the MVTec AD and VisA benchmark datasets with synthetic label noise (0% to 20%) demonstrate that MLG-STPM significantly improves anomaly detection and localization performance compared to the original STPM, especially under higher noise conditions, and achieves competitive results against other relevant approaches.

## 1. Introduction

Industrial image anomaly detection (IAD) plays a pivotal role in modern manufacturing, serving as a cornerstone for automated quality control and process optimization. It exists within the broader field of fault diagnosis, which encompasses a wide array of challenges, from detecting compound defects in mechanical systems using advanced signal processing and biologically inspired neural networks [1] to assessing the reliability of complex controllers by modeling multi-component failure dependencies [2]. Within this context, this paper focuses on a crucial sub-domain: the detection of surface anomalies such as scratches, cracks, contaminations, or structural imperfections in products ranging from textiles and metals to semiconductors. This task is vital for maintaining product quality, reducing waste, and ensuring operational safety [3,4,5,6]. Traditional manual inspection for such visual defects is often subjective, labor-intensive, and inefficient, especially in high-throughput production lines, motivating the shift towards automated visual inspection systems [3].

Recent advancements in deep learning have significantly propelled the field of IAD. Particularly relevant are unsupervised anomaly detection (UAD) methods, which learn the distribution of normal data exclusively from anomaly-free samples and identify anomalies as deviations from this learned normality [5]. This paradigm is highly practical for industrial settings where anomalous samples are typically rare, diverse, and expensive to collect and label exhaustively [3]. A comprehensive IAD system must perform two core tasks: image-level classification, to determine whether an anomaly is present, and pixel-level localization, to identify the exact location of defects [7,8]. The former provides a binary determination of product conformity, while the latter is crucial for root cause analysis.

UAD approaches have primarily evolved along two main branches. The first consists of reconstruction-based methods, utilizing models like Autoencoders and GANs to learn the normal data manifold [9,10,11]. The second and often more powerful branch involves feature embedding-based methods. These techniques map normal samples into a compact feature space using pretrained networks, one-class classification, or memory banks [12,13,14,15,16]. Within this latter category, Student–Teacher Feature Pyramid Matching (STPM) has emerged as a state-of-the-art technique [17,18]. STPM leverages knowledge distillation, where a pretrained teacher network provides descriptive features for normal samples, and a student network learns to match these features. Anomalies are then detected by significant discrepancies between the student’s and teacher’s feature representations [17].

However, a critical challenge arises when applying UAD methods like STPM. They operate under the implicit assumption that the training dataset consists solely of pristine anomaly-free samples. In practice, industrial training data are frequently contaminated with label noise—where anomalous samples are mistakenly included in the normal training set due to annotation errors or evolving defect definitions [3,19,20,21]. Such contamination leads the model to learn a compromised representation of normality, resulting in a blurred decision boundary and severely degraded detection performance [21]. Recognizing this critical issue, several recent studies have begun to explicitly tackle the challenge of label noise in UAD, proposing specialized techniques such as robust patch-level modeling [21] or sample re-weighting schemes.

To address this, we turn to the field of Learning with Noisy Labels (LNL). A foundational discovery in LNL is the memorization effect of deep neural networks: they tend to first learn simple prevalent patterns (clean samples) before eventually overfitting to noisy samples [22]. This insight gives rise to the “small-loss criterion” [23], a heuristic for identifying reliable samples based on their low training loss. Meta-learning offers a powerful framework to operationalize such principles for robust training. However, conventional meta-learning approaches typically rely on a small clean “meta-set” of trusted data to guide the learning process [24]. This requirement presents a fundamental conflict within the noisy UAD setting, where, by definition, such an externally-verified clean dataset is unavailable.

This specific challenge directly motivates our work. We propose Meta-Learning Guided STPM (MLG-STPM), a framework designed to adapt meta-learning principles to the constraints of the noisy UAD problem. Our core contribution is the Evolving Meta-Set (EMS), a mechanism that resolves the aforementioned conflict by dynamically constructing a reliable proxy for a clean meta-set from the noisy training data themselves. Leveraging the small-loss criterion, the EMS continuously curates a small buffer of high-confidence samples. By guiding the student network with this internally generated meta-set, MLG-STPM enhances the robustness of STPM without requiring an external clean dataset.

The main contributions of this work are as follows:

- We systematically analyze the detrimental effect of varying levels of label noise on the performance of the STPM unsupervised anomaly detection method.
- We propose a novel framework, MLG-STPM, which augments STPM with an Evolving Meta-Set (EMS) mechanism. This enhances robustness by dynamically generating a high-confidence set of training samples, thereby removing the dependency on an external clean dataset common in traditional meta-learning.
- We demonstrate through comprehensive experiments on the MVTec AD and VisA datasets that MLG-STPM consistently and significantly outperforms the baseline STPM under various noise conditions, achieving competitive results against other relevant approaches.

The remainder of this paper is organized as follows. Section 2 reviews the related work. Section 3 details the proposed MLG-STPM methodology. Section 4 presents the experimental setup and results. Section 5 provides further discussion, and Section 6 concludes the paper.

## 2. Related Work

This section provides a background on unsupervised anomaly detection, discusses student–teacher frameworks, and reviews relevant techniques from the field of LNL that form the theoretical foundation of our approach.

### 2.1. Unsupervised Anomaly Detection in Industrial Images

UAD aims to identify patterns that deviate significantly from the majority of the data, assuming access only to normal samples during training [3,5]. Traditional methods often relied on statistical approaches or classical machine learning, but these struggle with the high dimensionality and complexity of image data [3]. Deep learning has become dominant, primarily through two paradigms:

- Reconstruction-based Methods: These methods train models such as Autoencoders (AEs) [9], Variational Autoencoders (VAEs) [10], and GANs [11] to reconstruct normal images accurately. Anomalies are detected based on high reconstruction errors. While intuitive, controlling the model’s generalization to avoid reconstructing anomalies themselves remains a challenge.
- Feature Embedding-based Methods: These methods map images into a feature space using pretrained or specially trained networks [3,5]. Prominent techniques include one-class classification like Deep-SVDD [13] and memory-bank-based approaches like PaDiM [14] and PatchCore [25]. Moreover, other significant paradigms such as normalizing flows [26,27] and diffusion probabilistic models [4] have demonstrated strong performance by modeling the distribution of normal features. Student–teacher frameworks, which we discuss next, are a particularly effective subclass within this broad category.

A common limitation of most UAD methods is their reliance on a clean training set, which restricts their applicability in real-world industrial settings where label noise is prevalent [3,21].

Addressing this limitation has recently become an active area of research. For instance, SoftPatch [21] introduces a mechanism to dynamically soften patch representations to mitigate the influence of anomalous samples. Other approaches have explored techniques like latent outlier exposure [20] or robust loss functions. Our work contributes to this growing subfield but takes a different perspective. Instead of designing a novel architecture from scratch, we focus on enhancing the robustness of an existing, state-of-the-art Student–Teacher framework (STPM), a direction that remains relatively underexplored.

### 2.2. Student–Teacher Frameworks for Anomaly Detection

The student–teacher paradigm, originating from knowledge distillation, has been successfully adapted for UAD [12,28]. In this setup, a pretrained, frozen teacher network provides rich feature representations for normal images. A student network is then trained to mimic the teacher’s output for these normal samples. The STPM method [17,18] is a prominent example, which has itself been extended with reconstruction-based students [29]. Other distillation-based approaches, such as Reverse Distillation, also explore this paradigm from different perspectives [28]. While effective on clean data, STPM’s performance degrades significantly when the training set contains mislabeled anomalies, as the student network incorrectly learns these anomalous patterns as normal.

### 2.3. Robust Learning via Sample Selection

To address the challenge of label noise, our work draws upon established principles from the field of LNL.

Memorization Effect and the Small-Loss Criterion. A key observation in LNL is the memorization effect of deep neural networks. High-capacity networks first learn the simple underlying patterns present in the majority of the data, which predominantly consist of clean samples, before eventually memorizing the noisy labels of individual corrupted samples [22]. This behavior provides a critical window of opportunity for robust training, implying that during the early-to-intermediate training stages, samples exhibiting a smaller loss are highly likely to be clean. This principle, known as the “small-loss criterion,” has become a cornerstone for many sample selection methods in LNL [23]. These methods typically select a subset of small-loss samples to guide the training, thereby mitigating the negative impact of noise. To ensure these loss values are meaningful, a warm-up period is often employed. During this phase, the model trains on the entire noisy dataset to stabilize from its initial random state and begin capturing the dominant data patterns.

Meta-Learning and Sample Weighting. Meta-learning offers a more sophisticated approach to LNL, aiming to “learn how to learn” from noisy data. Methods like CMW-Net [24], for example, employ a complex bi-level optimization scheme where a small clean meta-dataset is used to train a meta-network that learns to assign optimal weights to training samples. This sample selection and weighting approach is one of the two main paradigms for handling label noise. The other prominent paradigm is loss correction, which directly adjusts the loss function for each sample. For instance, some work has proposed theoretically grounded importance reweighting frameworks that can be applied to any surrogate loss function to correct for class-conditional noise [30]. While powerful, these approaches often introduce significant computational overhead and rely on the accurate estimation of noise rates. Our MLG-STPM is inspired by the meta-learning goal of adaptively guiding the learning process, but we consciously opt for a more direct and efficient implementation based on the small-loss criterion, avoiding the complexity of explicit weight-learning or bi-level optimization.

Distinction from Inference–Time Memory Banks. It is crucial to distinguish our training-time EMS from the memory banks utilized in methods like PatchCore [25]. A PatchCore memory bank, or coreset, is constructed once from the training data and serves as a static database of normal features for inference. Anomaly scores are then computed by comparing a test sample’s features against this fixed reference set. In stark contrast, our EMS is a dynamic component integral to the training process itself. Its purpose is not to score test samples but to provide a continuously refined high-confidence subset of training data to anchor and stabilize the student network’s learning. Thus, while both approaches involve curating a data subset, our EMS functions as a guidance mechanism for robust training, whereas a conventional memory bank acts as a reference for inference-time scoring.

## 3. Methodology

This section presents the methodology of our proposed MLG-STPM framework. Our approach enhances the standard STPM architecture with a novel guidance mechanism to address the challenge of label noise in UAD. For clarity and easy reference, a comprehensive list of all symbols and their definitions used throughout this section is provided in Appendix A. We first provide a brief overview of the STPM baseline. Then, we introduce our two-stage training strategy, detailing the rationale behind the initial warm–up phase and the subsequent operation of our core contribution, the EMS.

### 3.1. Preliminaries: Student–Teacher Feature Pyramid Matching

**Figure 1:**
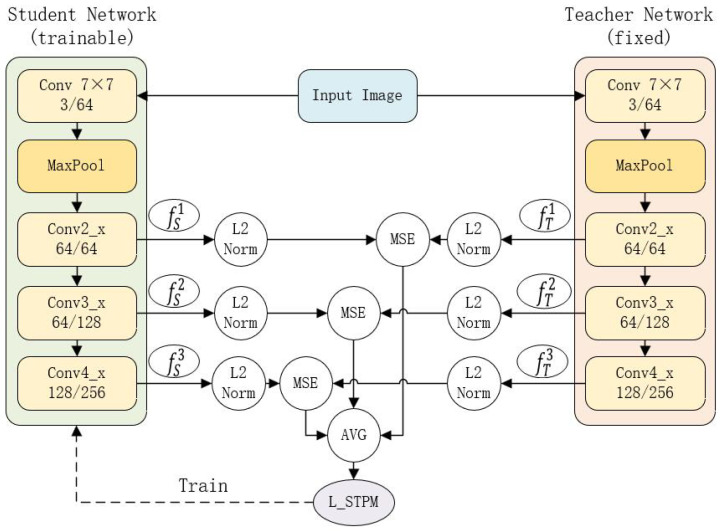
Illustration of the STPM baseline architecture. Both the fixed teacher and trainable student networks share a ResNet-18 backbone, with feature matching performed on intermediate layers (Conv2_x, Conv3_x, Conv4_x). The solid lines indicate the forward data flow, while the dashed line represents the backpropagation of the training loss. The Mean Squared Error (MSE) between the L2-normalized feature maps of the two networks constitutes the final training loss, $L_{\mathrm{STPM}}$.

Our method is built upon the STPM framework [17], a powerful paradigm for unsupervised anomaly detection. This approach utilizes two deep convolutional neural networks: a pretrained fixed teacher network, denoted as *T*, and a trainable student network, denoted as *S*. Both networks typically share an identical architecture, such as ResNet, to facilitate effective knowledge transfer. The architecture of this feature pyramid matching process, which is based on a ResNet-18 backbone as in the original STPM, is illustrated in Figure 1.

For an input image $I\in R^{H\times W\times C}$, both networks extract feature maps from a set of *L* corresponding intermediate layers. For the *l*-th layer (l∈{1,…,L}), the teacher network outputs a feature map $f_{T}^{l}\left(I\right)\in R^{H_{l}\times W_{l}\times C_{l}}$, and the student network outputs $f_{S}^{l}\left(I;\theta_{S}\right)\in R^{H_{l}\times W_{l}\times C_{l}}$, where $\theta_{S}$ are the learnable parameters of the student network.

To focus on the directional differences in the feature space, these feature maps are L2-normalized:

(1) $${\hat{f}}^{l}\left(I\right)=\frac{f^{l}\left(I\right)}{∥f^{l}\left(I\right){∥}_{2}}.$$

The training objective is to minimize the discrepancy between the student’s and teacher’s normalized feature maps, thereby teaching the student the distribution of normal data. The per-sample loss, representing this discrepancy, is key to our method and is defined as

(2) $$L_{\mathrm{sample}}\left(I_{k};\theta_{S}\right)=\frac{1}{L}\sum_{l=1}^{L}{∥{\hat{f}}_{T}^{l}\left(I_{k}\right)-{\hat{f}}_{S}^{l}\left(I_{k};\theta_{S}\right)∥}_{2}^{2}.$$

### 3.2. Proposed Method: MLG-STPM

**Figure 2:**
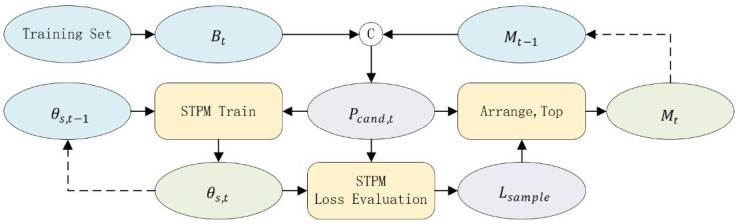
The data flow of our proposed MLG-STPM training strategy. At each iteration *t*, a candidate pool ($P_{\mathrm{cand},t}$) is formed by combining the current mini-batch ($B_{t}$) and the previous meta-set ($M_{t-1}$). The student network, with parameters $\theta_{s,t-1}$, is trained on these data. The resulting per-sample loss ($L_{\mathrm{sample}}$) is then used to evaluate the candidate pool and select the new high-confidence meta-set ($M_{t}$), creating a robust feedback loop that updates the student parameters to $\theta_{s,t}$. Solid lines represent the main data flow within an iteration, while dashed lines indicate updates between iterations.

The standard STPM’s effectiveness is compromised by label noise. To counter this, we introduce MLG-STPM, a framework that integrates a robust training strategy inspired by principles from LNL. Our method operates in two distinct stages: a warm-up phase to enable reliable loss-based sample evaluation, followed by an EMS-guided training phase. The data flow of our proposed training strategy is depicted in Figure 2.

#### 3.2.1. Stage 1: Warm-Up for Reliable Loss Evaluation

At the beginning of training, the student network’s parameters $\theta_{S}$ are randomly initialized. Consequently, the per-sample loss $L_{\mathrm{sample}}$ is not yet a meaningful indicator of a sample’s normality; both normal and anomalous samples will produce large and unpredictable losses. Applying the small-loss criterion at this point would lead to the selection of a random unreliable set of samples.

To address this, we institute a warm-up phase for the first $N_{\mathrm{warmup}}$ training batches. During this phase, the student network is trained using the standard STPM objective on the entire training set, which is composed of a majority of normal samples and a minority of anomalous ones, without any sample selection. According to the memorization effect [22], this initial training forces the network to first learn the simpler dominant patterns corresponding to the majority class of normal samples. After this warm-up, the student network has developed a preliminary understanding of the normal data manifold, and the loss $L_{\mathrm{sample}}$ becomes a reliable proxy for sample cleanliness: normal samples tend to yield lower losses than anomalous ones.

#### 3.2.2. Stage 2: EMS-Guided Robust Training

Following the warm-up phase, we activate our core mechanism, the EMS. This mechanism employs a dynamic buffer, denoted as M, with a fixed capacity $K_{\mathrm{meta}}$. Its purpose is to store a collection of high-confidence normal samples, thereby operationalizing the small-loss criterion [23].

##### EMS Update

At each training iteration *t* (where $t>N_{\mathrm{warmup}}$), the EMS is updated through a two-step process. First, we form a candidate pool, $P_{\mathrm{cand}}$, by combining the images from the current mini-batch $B_{t}$ with all images from the previous EMS iteration, $M_{t-1}$:

(3) $$P_{\mathrm{cand},t}=B_{t}\cup M_{t-1}.$$

Next, we evaluate every sample within this pool by calculating its per-sample loss (Equation (2)) using the current student parameters, $\theta_{S,t}$. The new EMS, $M_{t}$, is then populated by selecting the $K_{\mathrm{meta}}$ images from the candidate pool that exhibit the lowest losses:

(4) $$M_{t}=\underset{I_{k}\in P_{\mathrm{cand},t}}{\arg\;\mathrm{top}\text{-}K_{\mathrm{meta}}}(-L_{\mathrm{sample}}\left(I_{k};\theta_{S,t}\right)).$$

This iterative select-and-replace mechanism ensures that M always contains the most reliable training instances available, acting as a stable anchor for the learning process.

##### EMS-Guided Student Learning

To leverage the EMS for robust training, we construct an augmented training batch $B_{t}^{\prime }$ by combining the current mini-batch $B_{t}$ with the high-confidence images from the EMS:

(5) $$B_{t}^{\prime }=B_{t}\cup M_{t-1}.$$

The student network is then updated by minimizing the guided training loss $L_{\mathrm{guided}}$, which is the average per-sample loss over this augmented batch:

(6) $$L_{\mathrm{guided}}\left(B_{t}^{\prime };\theta_{S}\right)=\frac{1}{|B_{t}^{\prime }|}\sum_{I_{k}\in B_{t}^{\prime }}L_{\mathrm{sample}}\left(I_{k};\theta_{S}\right).$$

The parameters are updated via stochastic gradient descent:

(7) $$\theta_{S,t}=\theta_{S,t-1}-\alpha \nabla_{\theta_{S}}L_{\mathrm{guided}}\left(B_{t}^{\prime };\theta_{S,t-1}\right).$$

By consistently training on this augmented batch, which is enriched with reliable samples from the EMS, the student network’s learning is continuously stabilized, preventing it from overfitting to noisy samples that may appear in any single mini-batch. The complete procedure is outlined in Algorithm 1.

**Algorithm 1** MLG-STPM Training Process**Require:** Pretrained teacher network *T*; student network *S* with initial parameters $\theta_{S,0}$;    training dataset *D*; mini-batch size $N_{B}$; meta-set size $K_{\mathrm{meta}}$; warm-up iterations $N_{\mathrm{warmup}}$;    learning rate α.
  1:Initialize EMS $M_{0}=\emptyset$.  2:Initialize iteration counter t=0.  3:**for** each epoch **do**  4:    **for** each mini-batch $B_{t}\subset D$ **do**  5:        t←t+1.  6:        **if** $t>N_{\mathrm{warmup}}$ **then**  7:           Construct augmented batch $B_{t}^{\prime }$ using Equation (5) with $M_{t-1}$.  8:        **else**  9:           $B_{t}^{\prime }\leftarrow B_{t}$.               ▹ Warm-up phase: use original batch10:        **end if**11:        Compute guided loss $L_{\mathrm{guided}}\left(B_{t}^{\prime };\theta_{S,t-1}\right)$ using Equation (6).12:        Update student parameters to obtain $\theta_{S,t}$ using Equation (7).13:        **if** $t>N_{\mathrm{warmup}}$ **then**             ▹ Activate EMS update after warm-up14:           Form candidate pool $P_{\mathrm{cand},t}$ using Equation (3) with $B_{t}$ and $M_{t-1}$.15:           Update EMS to obtain $M_{t}$ from $P_{\mathrm{cand},t}$ according to Equation (4).16:        **else**17:           $M_{t}\leftarrow M_{t-1}$.            ▹ Keep EMS unchanged during warm-up18:        **end if**19:    **end for**20:**end for**21:**return** Final trained student parameters $\theta_{S}^{*}$.


### 3.3. Inference and Anomaly Scoring

During inference, the EMS and its associated mechanisms are inactive. Given a test image *J*, anomaly detection follows the standard STPM procedure. This process generates two complementary outputs for a comprehensive anomaly assessment: a dense map for localization and a single score for classification.

1. Feature Discrepancy Calculation: For each layer *l*, a per-layer anomaly map $A^{l}\left(J\right)$ is computed. The score at each spatial location (h,w) is the cosine distance between the teacher’s and student’s normalized feature vectors:
  (8) $${\left(A^{l}\left(J\right)\right)}_{h,w}=1-\frac{{\hat{f}}_{T}^{l}{\left(J\right)}_{h,w}\;\cdot \;{\hat{f}}_{S}^{l}{\left(J;\theta_{S}^{*}\right)}_{h,w}}{∥{\hat{f}}_{T}^{l}{\left(J\right)}_{h,w}{∥}_{2}{∥{\hat{f}}_{S}^{l}{\left(J;\theta_{S}^{*}\right)}_{h,w}∥}_{2}},$$
  where $\theta_{S}^{*}$ represents the final trained parameters of the student network, and ${\left(\;\cdot \;\right)}_{h,w}$ denotes the feature vector at spatial coordinates (h,w).
2. Pixel-level Anomaly Map Generation: Each per-layer map $A^{l}\left(J\right)$ is up-sampled to the input image resolution, H×W, and subsequently aggregated via element-wise multiplication. This yields the final anomaly map, Ω(J).
  (9) $$\Omega \left(J\right)=\prod_{l=1}^{L}\mathrm{Upsample}\left(A^{l}\left(J\right)\right)$$
  This map, Ω(J), directly constitutes the output for the pixel-level localization task.
3. Image-level Score Derivation: While the anomaly map provides detailed spatial information, a single scalar value is required for the subsequent image-level classification task. Therefore, we derive a final score for image *J* by taking the maximum value from the pixel-level anomaly map:
  (10) $$\mathrm{Score}\left(J\right)=\max_{h,w}{\left(\Omega \left(J\right)\right)}_{h,w}.$$
  This derivation establishes the connection between the two outputs, as the image-level score is a function of the most salient anomaly at the pixel level.

Thanks to the EMS-guided training, the student network $S(\theta_{S}^{*})$ provides a more robust representation of normality, leading to more accurate pixel-level localization and image-level classification, especially in noisy environments.

## 4. Experiments

### 4.1. Datasets and Evaluation Setup

Our experimental setup is designed following the principles of the IM-IAD benchmark [8], which provides a uniform framework for evaluating industrial IAD algorithms under various realistic conditions. We conduct our experiments on two widely-used public benchmarks featured in the IM-IAD framework. A statistical summary is provided in Table 1.

**Figure 3:**
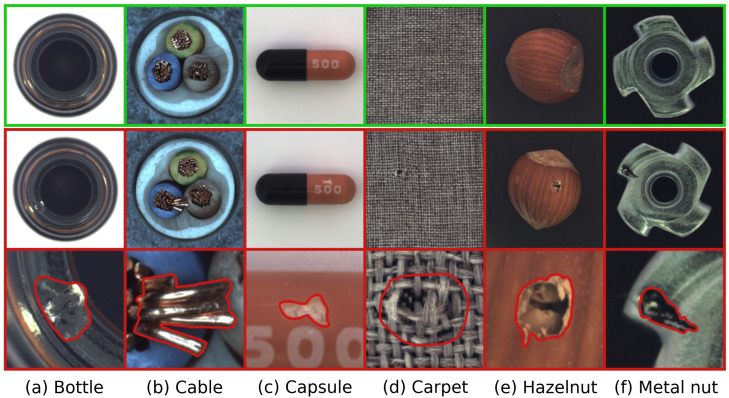
Examples of normal and anomalous samples from the MVTec AD dataset. The **top row** illustrates normal pristine items. The **middle row** presents anomalous counterparts, while the **bottom row** provides close-up views of these anomalies, often highlighted with contours to emphasize the defect regions. This selection demonstrates the diverse defect types encountered across various object and texture categories within the dataset.
- VisA [3]: A more recent and challenging dataset designed to reflect complex industrial inspection scenarios. It contains 10,621 normal and 1200 anomaly images across 12 object categories. The dataset is particularly difficult as it includes images with multiple objects and lacks consistent camera alignment, posing additional challenges for anomaly detection algorithms. Representative samples illustrating these complexities are shown in Figure 4.

- MVTec AD [7,31]: A foundational benchmark for unsupervised anomaly detection. It comprises 5 texture and 10 object categories, totaling 3629 normal images for training and 1725 images for testing, comprising 467 normal and 1258 anomalous instances. It covers over 70 distinct artificially induced defect types, as illustrated in Figure 3.

**Figure 4:**
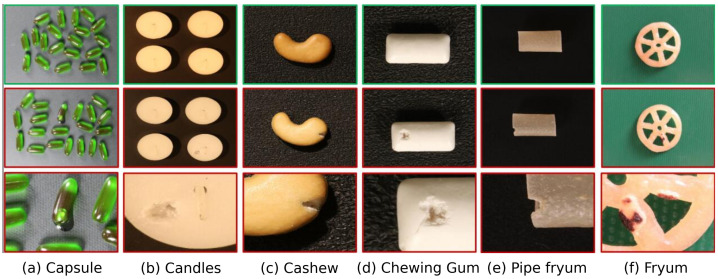
Representative samples from the VisA dataset. The **top row** showcases various normal instances. The **middle row** displays anomalous counterparts, and the **bottom row** offers detailed zoomed-in views of the defects, frequently outlined with contours to highlight the anomalous regions. This illustrates the complexity and variability of anomalies in this challenging multi-object dataset.

#### 4.1.1. Noisy IAD Setting

To evaluate model robustness against contaminated data, we adopt the Noisy IAD setting from the IM-IAD benchmark. In this paradigm, the training data are polluted with anomaly samples that are mislabeled as normal, simulating common annotation errors in industrial settings. To practically implement this for datasets like MVTec AD and VisA, where the provided training sets are pristine, we synthetically create noisy training sets.

The process involves randomly sampling a subset of anomaly images from the original test set and adding them to the normal-only training set. The noise ratio, η, is defined as the number of added anomaly samples divided by the number of original normal training samples. We evaluate performance under various noise ratios: η∈{0,0.05,0.10,0.15,0.20}, corresponding to 0% to 20% noise contamination.

#### 4.1.2. Evaluation Protocol

To ensure a fair and rigorous evaluation in line with standard scientific practice for this setup, we adhere to the no-overlap protocol. Under this protocol, any anomaly samples that are moved into the training set for noise injection are subsequently removed from the test set. This prevents the model from being tested on the same anomalous instances it was exposed to as normal samples during training, thereby providing a true measure of its generalization capability on unseen defects.

### 4.2. Implementation Details

To ensure reproducibility and comparability, we detail the common experimental settings and hyperparameters used across all evaluated methods. For each baseline method, we either adopted the recommended configurations from their original publications or carefully tuned them for optimal performance on the clean dataset. Our proposed MLG-STPM method shares the same hyperparameters as the STPM baseline for a fair comparison, unless otherwise specified. The capacity of the Evolving Meta-Set, $K_{\mathrm{meta}}$, is an important parameter. Its value is chosen to balance two competing factors: it must be large enough relative to the mini-batch size to provide a stable guidance signal against noise, yet not so large as to risk overfitting by limiting exposure to new training samples. We found a small multiple of the mini-batch size to be an effective choice that satisfies this principle. Table 2 summarizes the key training parameters for the methods compared in this study.

### 4.3. Evaluation Metrics

To comprehensively evaluate our method’s performance on the two core tasks defined in the introduction, namely image-level classification and pixel-level localization, we adopt the following standard metrics in line with established benchmarks [31].

- Area Under the ROC Curve (AU-ROC): This metric evaluates a model’s ability to distinguish between normal and anomalous instances. We report this metric for both tasks. For image-level classification, the AU-ROC is computed from the final anomaly score of each image (I-AUROC). For pixel-level localization, it is computed from the pixel-wise anomaly map (P-AUROC). The formula is
  (11) $$\mathrm{AU}\text{-}\mathrm{ROC}=\int_{0}^{1}\mathrm{TPR}\;d\left(\mathrm{FPR}\right),$$
  where TPR and FPR are the True Positive Rate and False Positive Rate, respectively.
- Area Under the Precision-Recall Curve (AU-PR): This metric is particularly robust for imbalanced datasets. It is also applied to both the final image scores (I-AP) and the pixel-wise maps (P-AP). For consistency with related literature, we refer to it as Average Precision (AP) in our result tables. Its conceptual formula is
  (12) $$\mathrm{AU}\text{-}\mathrm{PR}=\int_{0}^{1}P\left(R\right)\;dR,$$
  where P(R) denotes precision as a function of recall.
- Area Under the Per-Region Overlap Curve (AUPRO): This metric specifically assesses segmentation quality. As it inherently measures spatial accuracy, it is exclusively used to evaluate pixel-level localization performance. AUPRO integrates the Per-Region Overlap (PRO) metric over multiple thresholds:
  (13) $$\mathrm{PRO}=\frac{1}{N}\sum_{i=1}^{N}\frac{|P_{i}\cap C_{i}|}{|C_{i}|},$$
  where $C_{i}$ is the *i*-th ground-truth anomaly region, $P_{i}$ is the set of correctly predicted anomalous pixels within that region, and *N* is the total number of ground-truth regions.

For all metrics, a higher value indicates better performance, and all reported scores are averaged across the respective dataset categories.

### 4.4. Results and Analysis

This section presents the quantitative results, first comparing MLG-STPM with other methods under a fixed noise setting, then analyzing its robustness against the baseline across various noise levels.

#### 4.4.1. Comparative Results

We first evaluate our method against other approaches in a typical noisy scenario with a 10% noise ratio (η=0.10). The results are presented in Table 3.

As shown in Table 3, on MVTec AD, MLG-STPM achieves highly competitive results, leading in P-AUROC, I-AUROC, P-AP, I-AP, and P-AUPRO metrics, and outperforming most listed methods across these measures. Notably, its strong P-AUPRO performance indicates superior localization capability, surpassing both Reverse Distillation and the STPM baseline. On the more challenging VisA dataset, MLG-STPM also demonstrates strong performance. While the STPM baseline achieves the highest P-AUROC, MLG-STPM secures the best performance in several other key metrics, including I-AUROC, P-AP, I-AP, and P-AUPRO, establishing itself as a robust and effective method in a noisy UAD setting.

#### 4.4.2. Robustness to Varying Label Noise

The core contribution of our work is enhancing robustness against different levels of label noise. Table 4 presents a detailed comparison of MLG-STPM against the STPM baseline across noise ratios from 0% to 20%.

On MVTec AD, the effectiveness of the EMS mechanism is striking. Even in the clean setting (η=0.00), MLG-STPM significantly improves the I-AUROC compared to STPM. As noise is introduced, the baseline STPM’s performance generally degrades across metrics, with its P-AUPRO showing a noticeable decline at higher noise levels. In stark contrast, MLG-STPM maintains an exceptionally stable and high performance, with its P-AUPRO remaining remarkably consistent across the entire noise spectrum, demonstrating striking robustness.

On VisA, a similar trend is observed. The baseline STPM’s performance deteriorates notably under noise, with its P-AUROC exhibiting a significant drop from 0% to 20% noise. Conversely, MLG-STPM exhibits much greater stability, with its P-AUROC and other metrics remaining largely constant across the entire noise spectrum. This stability is a key advantage in real-world scenarios where label noise is prevalent. The consistent and significant performance gap in most metrics between MLG-STPM and STPM, which tends to widen as noise increases, provides strong evidence for the efficacy of the proposed EMS mechanism.

#### 4.4.3. Qualitative Results

**Figure 5:**
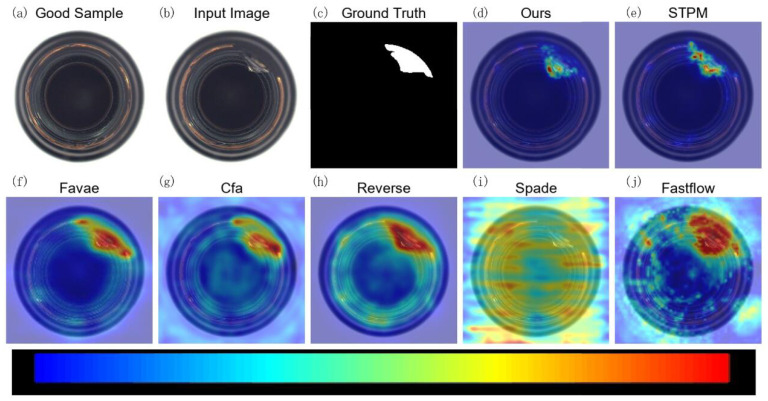
Qualitative anomaly detection results for a sample from the Capsule category (MVTec AD) under 10% label noise. The figure compares the anomaly heatmap generated by our MLG-STPM against the STPM baseline and other relevant methods.

**Figure 6:**
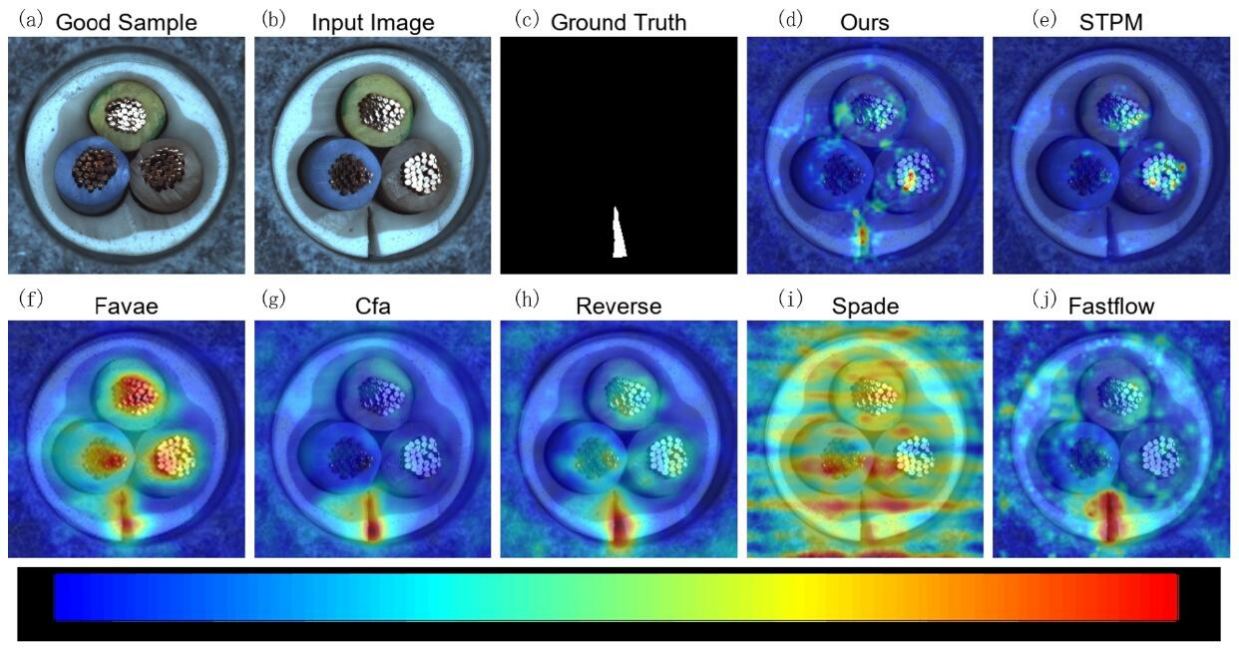
Qualitative anomaly detection results for the Cable category of the MVTec AD dataset under 10% label noise. This example highlights MLG-STPM’s superior localization and more concentrated anomaly response compared to baseline STPM and other methods for a different defect type.

**Figure 7:**
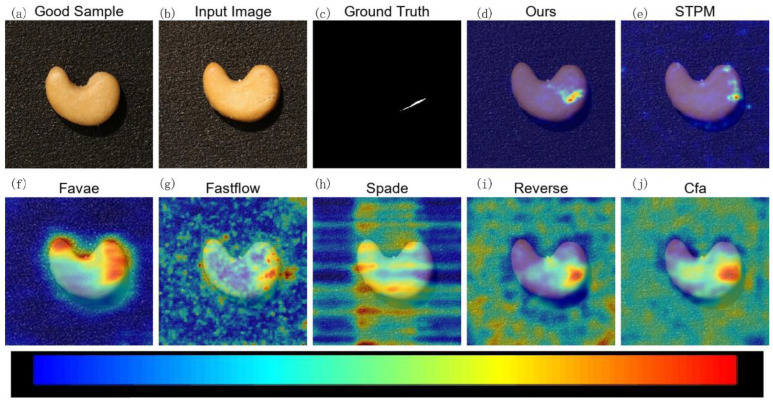
Qualitative anomaly detection results for the Chew category of the VisA dataset under 10% label noise. MLG-STPM effectively highlights the anomalous regions with high precision, demonstrating strong localization capabilities even with noisy training data.

**Figure 8:**
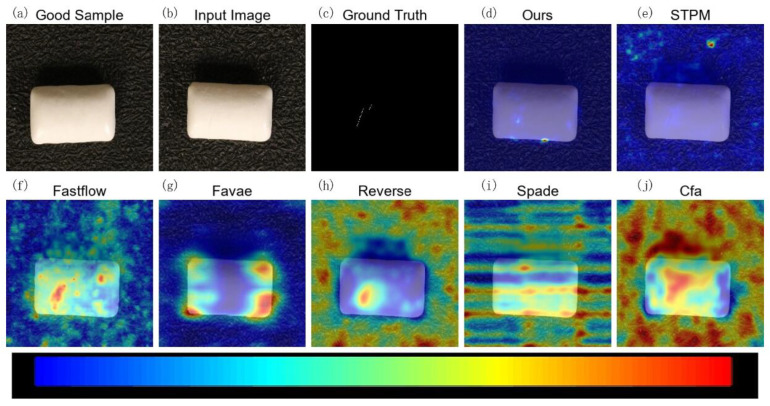
Qualitative anomaly detection results for the Pill category of the VisA dataset under 10% label noise. This figure illustrates MLG-STPM’s robustness and accuracy in localizing a subtle scratch defect on a pill surface with minimal false positives.

To visually demonstrate the effectiveness of MLG-STPM in localizing anomalies, we present qualitative anomaly maps for selected examples from the Motec AD and VisA datasets. In the following figures (Figure 5, Figure 6, Figure 7 and Figure 8), anomaly heatmaps are provided for visual comparison. For these heatmaps, the scores are normalized per image to highlight the areas of highest deviation from normality. The color scale is consistent across all figures: warmer colors (e.g., red, yellow) represent higher relative anomaly scores, indicating a higher likelihood of a defect, while cooler colors (e.g., blue, green) correspond to lower scores and normal regions. We first discuss the results for MVTec AD.

For the MVTec AD dataset, as demonstrated in Figure 5, the baseline STPM provides a reasonable anomaly map for the capsule anomaly. However, MLG-STPM consistently generates a more focused and intense highlight over the defect, aligning more accurately with the ground truth. Other methods often yield more diffuse or less accurate heatmaps, indicating a broader spread of anomaly scores that may encompass normal regions.

Figure 6, representing the Cable category, further illustrates MLG-STPM’s exceptional ability to precisely localize small and subtle defects. Compared to the baseline STPM, which often produces scattered or weaker anomaly responses, MLG-STPM consistently delivers a clearer and more concentrated anomaly map, a critical advantage for practical industrial inspection.

On the more challenging VisA dataset, MLG-STPM maintains its strong performance.

Figure 7 shows a complex anomaly on a sample from the Chew category. While STPM successfully detects the anomaly, MLG-STPM provides a superior anomaly map that is not only clearer but also more concentrated on the actual defect region, thereby minimizing false positives in surrounding normal areas. This precision is particularly crucial in reducing inspection errors.

Furthermore, Figure 8, depicting a Pill sample with a very subtle scratch, highlights MLG-STPM’s robust localization capabilities. It distinctly identifies the thin scratch, whereas many other methods either fail to detect such fine details or generate broader noisier anomaly maps that could lead to ambiguous interpretations. These consistent improvements in anomaly mapping, especially under noisy training conditions, are directly attributable to MLG-STPM’s Evolving Meta-Set (EMS) mechanism, which continuously guides the student network towards learning from a more reliable representation of normal data. This leads to anomaly maps that are not only accurate but also visually interpretable and actionable for quality control.

The qualitative results visually corroborate the quantitative findings, affirming the improved anomaly localization and reduced false detections achieved by MLG-STPM through its noise-robust training.

## 5. Discussion

The experimental results compellingly demonstrate that the proposed MLG-STPM framework significantly enhances the robustness of STPM against label noise. The efficacy of our approach is rooted in its principled application of concepts from Learning with Noisy Labels. The core two-stage mechanism—a warm-up phase followed by EMS-guided training—proves effective in mitigating the detrimental impact of noisy instances. This is achieved without requiring external clean data or complex meta-weighting schemes, highlighting its practicality.

Performance Analysis and Robustness. On MVTec AD, MLG-STPM shows clear and consistent superiority over the baseline STPM across all tested noise levels (0% to 20%). Its performance stability, even at 20% noise, is particularly noteworthy and directly attributable to the EMS providing a constant stream of reliable guidance. On the more challenging VisA dataset, MLG-STPM also demonstrates substantially improved robustness, especially in maintaining stable AUROC and AUPRO scores as noise increases, while the baseline’s performance degrades sharply. This underscores the strength of our method in AUROC and AUPRO under noise, which are arguably more critical for a reliable detection system than metrics like AP that can be more sensitive to score distributions. The occasional lower AP on VisA could be due to its high intra-class variance, where our simple small-loss criterion, while robust, may not perfectly align with maximizing precision for every subtle defect type.

Connection to Theory and Competitiveness. The success of MLG-STPM validates our central hypothesis: the memorization effect can be effectively leveraged in the UAD context. The warm-up phase successfully creates the conditions for the small-loss criterion to become a reliable indicator of normality, and the EMS acts as a simple yet powerful mechanism to operationalize this criterion. In comparison to other methods, MLG-STPM establishes itself as a highly competitive approach in the noisy UAD landscape. Its strength lies in its simplicity and directness, contrasting with more complex meta-learning methods while achieving strong empirical results.

Limitations and Future Work. A key limitation of this study is its reliance on synthetic noise. While this approach ensures reproducibility and facilitates fair comparisons under established benchmarks, it may not fully capture the complexity of real-world noise distributions. The scarcity of public industrial datasets containing naturally occurring and verified label noise, often due to data privacy and proprietary concerns, necessitates the use of such simulations in academic research.

A primary difference is that real industrial noise may exhibit systematic patterns rather than being purely random. For instance, anomalous samples erroneously labeled as normal often possess subtle defects, making them visually more similar to the normal class compared to randomly selected anomalies. Although our method demonstrates strong performance under the standard controlled conditions, its future validation on datasets with inherent organic label noise remains a crucial step to fully ascertain its real-world applicability.

An interesting avenue for future work is the development of more sophisticated synthetic noise protocols that better emulate these challenging cases. One potential strategy involves using a pre-trained model to identify and select anomalous samples that yield the lowest anomaly scores. Injecting these hard-to-detect samples as noise would create a more realistic testbed. However, such an approach could introduce a model-specific bias, complicating fair benchmarking and presenting a methodological challenge that warrants further investigation. The computational overhead for managing the EMS is modest, as it only involves loss calculation and sorting on a small candidate pool, making the trade-off for significant gains in robustness highly favorable in most industrial scenarios. Future work could also explore more adaptive EMS management strategies, such as dynamically adjusting the size of the meta-set ($K_{\mathrm{meta}}$) or incorporating more sophisticated selection criteria beyond just the loss value. Furthermore, on a broader note, a significant challenge for nuanced evaluation in this field is the common lack of fine-grained per-defect-type annotations in large-scale benchmarks. We advocate for the development of future public datasets that include such labels, as this would greatly facilitate more targeted analysis and the creation of specialized models for particularly critical industrial defect categories.

## 6. Conclusions

This paper addressed the critical challenge of label noise in unsupervised industrial anomaly detection. We proposed MLG-STPM, a novel framework that enhances the robustness of the STPM method by integrating a principled sample selection strategy inspired by the field of Learning with Noisy Labels. Our approach leverages the memorization effect of deep networks through a two-stage process: a crucial warm-up phase enables reliable sample evaluation, after which an EMS dynamically curates a small buffer of high-confidence samples, identified by their low loss, to robustly guide the student network’s learning.

Comprehensive experiments on the MVTec AD and VisA benchmarks with varying levels of synthetic label noise (0% to 20%) demonstrated that MLG-STPM significantly and consistently outperforms the baseline STPM, especially under high-noise conditions. The proposed method showed substantial improvements across key image-level and pixel-level metrics, particularly on MVTec AD, and notable robustness gains on VisA. It also achieved competitive performance against other state-of-the-art techniques, affirming its effectiveness.

By effectively adapting and implementing the well-established small-loss criterion for the UAD domain, MLG-STPM offers a practical, robust, and efficient approach to improving the resilience of student–teacher-based anomaly detection systems in realistic industrial settings. It provides a strong foundation for future research into noise-robust UAD, with potential avenues including more adaptive EMS management and validation on a wider range of real-world noisy datasets.

## Figures and Tables

**Table 1:** Statistical overview of the MVTec AD and VisA datasets. Data are aggregated from [3,7,8].

Dataset	Sample Number		Classes		Image Resolution	
	Normal	Anomaly	Anomaly Type	Object	Min	Max
MVTec AD	4096	1258	73	15	700	1024
VisA	10,621	1200	78	12	960	1562

**Table 2:** Key hyperparameters and training settings for compared methods.

Method	Training Epochs	Batch Size	Image Size	Learning Rate
CFA [32]	50	4	256	0.001
FastFlow [27]	500	32	256	0.001
FAVAE [10]	100	64	256	0.00001
Reverse [28]	200	8	256	0.005
SPADE [16]	1	8	256	N/A
STPM (baseline) [17]	100	8	256	0.4
MLG-STPM (Ours)	100	8	256	0.4

**Table 3:** Comparison with state-of-the-art methods on MVTec AD and VisA under 10% label noise (η=0.10). Best performance is in bold; second best is underlined.

Method	Dataset	P-AUROC	I-AUROC	P-AP	I-AP	P-AUPRO
CFA [32]	MVTec AD	0.9340	0.9817	0.5759	0.9948	0.8459
	VisA	0.8465	0.8673	0.1286	0.9010	0.7177
FastFlow [27]	MVTec AD	0.9499	0.9603	0.4909	0.9860	0.8347
	VisA	0.9518	0.8837	0.0605	0.8739	0.8730
FAVAE [10]	MVTec AD	0.9775	0.9881	0.7052	0.9965	0.9183
	VisA	0.9715	0.8422	0.1123	0.8470	0.9023
Reverse [28]	MVTec AD	0.9770	0.8944	0.6989	0.9690	0.9207
	VisA	0.9713	0.8375	0.1552	0.9037	0.9044
SPADE [16]	MVTec AD	0.6311	0.9889	0.0784	0.9963	0.2274
	VisA	0.6263	0.8447	0.0018	0.8682	0.1886
STPM (baseline) [17]	MVTec AD	0.9729	0.9738	0.6916	0.9924	0.9253
	VisA	**0.9750**	0.8932	0.1402	0.9025	0.8997
MLG-STPM (Ours)	MVTec AD	**0.9817**	**0.9929**	**0.7093**	**0.9976**	**0.9419**
	VisA	0.9723	**0.8953**	**0.1598**	**0.9085**	**0.9126**

**Table 4:** Performance of STPM and MLG-STPM on MVTec AD and VisA under varying label noise ratios (η). The proposed MLG-STPM demonstrates significantly greater robustness. The best performance for each comparison is highlighted in bold. Scores are averaged over all categories.

Dataset	Method	Noise (η)	P-AUROC	I-AUROC	P-AP	I-AP	P-AUPRO
MVTec AD	STPM (baseline)	0.00	0.9698	0.9238	0.6611	0.9777	0.9088
		0.05	0.9729	0.9913	0.6603	0.9974	0.9296
		0.10	0.9729	0.9738	0.6916	0.9924	0.9253
		0.15	0.9561	0.9460	0.5855	0.9845	0.8904
		0.20	0.9580	0.9698	0.5625	0.9910	0.8998
	MLG-STPM (Ours)	0.00	**0.9815**	**0.9929**	**0.7075**	**0.9976**	**0.9419**
		0.05	**0.9818**	**0.9937**	**0.7103**	**0.9979**	**0.9425**
		0.10	**0.9817**	**0.9929**	**0.7093**	**0.9976**	**0.9419**
		0.15	**0.9816**	**0.9921**	**0.7085**	**0.9973**	**0.9378**
		0.20	**0.9812**	**0.9929**	**0.7051**	**0.9976**	**0.9415**
VisA	STPM (baseline)	0.00	0.9675	**0.8943**	**0.1599**	**0.9015**	**0.9180**
		0.05	0.9619	**0.8830**	**0.1838**	**0.8939**	**0.9161**
		0.10	**0.9750**	0.8932	0.1402	0.9025	0.8997
		0.15	0.9692	**0.8810**	**0.1653**	**0.8952**	**0.9183**
		0.20	0.8941	0.8356	**0.1235**	0.8534	0.7844
	MLG-STPM (Ours)	0.00	**0.9716**	0.8734	0.1203	0.8742	0.9116
		0.05	**0.9719**	0.8765	0.1230	0.8776	0.9121
		0.10	0.9723	**0.8953**	**0.1598**	**0.9085**	**0.9126**
		0.15	**0.9717**	0.8732	0.1207	0.8741	0.9114
		0.20	**0.9721**	0.8727	0.1229	0.8729	0.9131

## Data Availability

The MVTec AD and VisA datasets analyzed during the current study are publicly available. MVTec AD can be found at https://www.mvtec.com/company/research/datasets/mvtec-ad (accessed on 6 October 2025), and VisA can be found at https://github.com/amazon-science/spot-diff (accessed on 6 October 2025). The code presented in this study is available on request from the corresponding author.

## Appendix A. List of Symbols

**Table A1:** List of symbols.

Symbol	Ref.	Description
*T*	Section 3.1	The pretrained and frozen teacher network.
*S*	Section 3.1	The trainable student network.
$\theta_{S}$	Section 3.1	Learnable parameters of the student network *S*.
I,J	Section 3.1	An input image from the training set (*I*) or test set (*J*).
H,W,C	Section 3.1	The height, width, and number of channels of an input image.
*l*	Section 3.1	Index for a specific layer in the network’s feature pyramid (l∈{1,…,L}).
*L*	Section 3.1	Total number of selected layers for feature extraction.
$f_{T}^{l}\left(I\right)$	Section 3.1	Feature map extracted from the *l*-th layer of the teacher network *T*.
$f_{S}^{l}\left(I;\theta_{S}\right)$	Section 3.1	Feature map extracted from the *l*-th layer of the student network *S*.
$H_{l},W_{l},C_{l}$	Section 3.1	The height, width, and number of channels of a feature map at layer *l*.
${\hat{f}}^{l}\left(I\right)$	Equation (1)	L2-normalized feature map.
${∥\;\cdot \;∥}_{2}$	Equation (1)	The L2-norm operation.
$L_{\mathrm{sample}}$	Equation (2)	The per-sample loss, used to evaluate a sample’s normality for EMS selection.
$N_{\mathrm{warmup}}$	Section 3.2.1	The number of warm-up iterations before activating the EMS.
M	Section 3.2.2	The Evolving Meta-Set (EMS), a dynamic buffer for high-confidence images.
$K_{\mathrm{meta}}$	Section 3.2.2	The fixed maximum capacity (a hyperparameter) of the EMS.
$P_{\mathrm{cand}}$	Equation (3)	The candidate pool for the EMS update, composed of the current mini-batch and the previous EMS.
$M_{t}$	Equation (4)	The state of the EMS at training iteration *t*.
$B_{t}$	Equation (3)	The current mini-batch of training data sampled from the training dataset at iteration *t*.
$\theta_{S,t}$	Equation (7)	The parameters of the student network *S* at iteration *t*.
$B_{t}^{\prime }$	Equation (5)	The augmented training batch at iteration *t*, composed of $B_{t}$ and $M_{t-1}$.
$L_{\mathrm{guided}}$	Equation (6)	The guided training loss for the student network, computed over $B_{t}^{\prime }$.
α	Equation (7)	The learning rate for the optimizer.
$\nabla_{\theta_{S}}$	Equation (7)	The gradient operator with respect to the student network’s parameters $\theta_{S}$.
$\theta_{S}^{*}$	Section 3.3	The final trained parameters of the student network.
$A^{l}\left(J\right)$	Equation (8)	The per-layer anomaly map generated from the feature discrepancy at layer *l*.
(h,w)	Equation (8)	Spatial coordinates within a feature map or image.
Ω(J)	Equation (9)	The final aggregated anomaly map for a test image *J*, used for pixel-level localization.
Score(J)	Equation (10)	The final image-level anomaly score for a test image *J*, used for image-level classification.
